# Pediatric road traffic accident – related head injury: a retrospective multicenter cohort study in Ghana

**DOI:** 10.1186/s12873-026-01687-5

**Published:** 2026-07-18

**Authors:** Anthony Baffour Appiah, Till Bärnighausen, Michael Lowery Wilson, Peter Dambach, Mahsa MohammadNamdar, Alexis Dun Bo-ib Buunaaim, Martin Tangnaa Morna, Vincent Ativor, Peter Donkor, Charles Mock

**Affiliations:** 1 Heidelberg Institute of Global Health, Faculty of Medicine and University Hospital, Heidelberg University, Heidelberg, Germany; 2 Section for Oral Health, Heidelberg Institute of Global Health, Faculty of Medicine and University Hospital, Heidelberg University, Heidelberg, Germany; 3 Injury Epidemiology and Prevention Research Group, Turku Brain Injury Centre, Department of Clinical Neurosciences, Turku University Hospital and University of Turku, Turku, Finland; 4 Department of Surgery, University for Development Studies, Tamale, Ghana; 5 Department of Trauma Orthopaedics, Tamale Teaching Hospital, Tamale, Ghana; 6 Department of Orthopedic and Trauma Surgery, Komfo Anokye Teaching Hospital, Kumasi, Ghana; 7 Department of Surgery, School of Medical Sciences, University of Cape Coast, Cape Coast, Ghana; 8 Department of Surgery, School of Medical Science, Kwame Nkrumah University of Science and Technology, Kumasi, Ghana; 9 Harborview Injury Prevention and Research Center, Harborview Medical Center, Seattle, USA

**Keywords:** Predictors, Glasgow outcome scale, Unfavorable outcome, Pediatric, Road traffic accident, Head injuries, Ghana

## Abstract

**Background:**

Head injuries (HIs) from road traffic accidents (RTAs) are major causes of death, long-term disability, and cognitive impairment in children. Evaluating significant predictors of functional outcomes after HI can help optimize trauma care and reduce the burden of unfavorable outcomes. This study examined the pre-hospital and clinical predictors of unfavorable outcomes in pediatric road traffic crash patients with HI (0–18 years).

**Methods:**

A retrospective cohort study of 816 pediatric RTA patients with HI presented at three teaching hospitals in the Northern, Ashanti, and Central Regions in Ghana. Functional outcome was assessed using the Glasgow Outcome Scale (GOS) at discharge, and categorized as either favorable (GOS 4–5) or unfavorable (GOS 1–3). A multivariable logistic regression model was used to identify predictors of unfavorable outcomes. The receiver operating characteristic (ROC) curve was used to assess the predictive performance of the multivariable logistic regression model. Kaplan-Meier failure analysis was used to estimate the cumulative incidence of HI-related inpatient mortality.

**Results:**

A total of 211 (25.9%) patients with HIs had an unfavorable inpatient outcome, including 45 (5.5%) deaths and 166 cases (20.3%) of severe disability. Significant predictors for higher odds of unfavorable outcomes were hospital location in the Northern Region (Adjusted odds ratio [AOR], 26.89; 95% confidence interval [CI], 10.87–66.53), patients with polytrauma (AOR = 1.94; 95% CI: 1.11–3.39) and those with unspecified head injuries (AOR = 2.08; 95% CI: 1.09–3.99), and ISS (AOR 1.04, 95% CI 1.00–1.07). The ROC curve showed a sensitivity of 69.52%, specificity of 94.81%, and an area under ROC curve of 0.9107. Kaplan-Meier analysis showed a higher cumulative probability of HI-related deaths for patients with severe injury (ISS > 15) and severe HI (GCS 3–8).

**Conclusion:**

Over one-quarter of pediatric RTA patients with HI in Ghana experienced unfavorable outcomes during hospitalization. Regional treatment location, greater injury severity, poly-trauma, and non-specific HI diagnoses independently predict unfavorable outcomes. These findings underscore the need to strengthen pediatric HI care through improved diagnostic capacity and early referral pathways, particularly for children with polytrauma and in the Northern Region.

**Supplementary Information:**

The online version contains supplementary material available at 10.1186/s12873-026-01687-5.

## Introduction

Head injuries (HIs) are a leading cause of injury-related deaths, long-term disability, and cognitive impairment in children worldwide [1, 2]. Pediatric patients with HI are at a higher risk of complications from head injuries compared to adults, due to differences in body size and anatomical proportions [3, 4]. Road traffic accidents (RTAs) are a major cause of head trauma and can result in a variety of extracranial and brain injuries, including deep scalp lacerations, skull fractures, cerebral hemorrhage, and brain contusions, among others [2, 5, 6]. More than 55% of HI in pediatric patients was caused by RTA worldwide [2, 6, 7]. Road crashes involving motorcycles, pedestrians, and bicycles remain the leading traffic-related mechanism of pediatric HI, especially in low- and middle-income countries (LMICs) [8, 9].

A common measure of both short-term and long-term functional outcomes in HI patients is the Glasgow Outcome Scale (GOS) [6, 10]. The GOS is a standardized assessment tool, and it is suitable for assessing functional outcomes following HI at hospital discharge, 30 days after discharge, 6 months after trauma, and at other time points relevant to the study context [6, 10].

Previous studies have documented several factors that directly or indirectly influence the functional outcomes of HI patients across different countries [2, 6, 7, 11, 12]. Significant predictors of unfavorable outcomes, as reported in studies from other countries, include age, mechanism of injury, severity of head injury, and emergency department vital signs such as Glasgow Coma Scale score, pupillary light reflex, heart rate, and blood pressure, among others [2, 6, 7, 11, 12]. Previous studies in Ghana examined the association between HI, sociodemographic, and pre-clinical factors in motorcycle crashes, as well as RTA-related mortality [7, 8, 13–15]. However, there are limited studies from Ghana that have comprehensively examined predictors of functional outcomes among pediatric patients.

A holistic assessment of prehospital and clinical predictors is critical to understanding how patient, crash, injury, and inpatient factors contribute to unfavorable outcomes in pediatric patients with RTA-related HI. Such evidence can inform prehospital and inpatient trauma care strategies to optimize outcomes for this vulnerable population. Therefore, this study examined the pre-hospital and inpatient predictors of unfavorable outcomes among pediatric RTA patients with HI at three teaching hospitals across ecological zones in Ghana.

## Methods

### The study design, setting, and population

This was a retrospective cohort study among pediatric patients who sustained road traffic-related head injuries and presented to the emergency departments (EDs) of three teaching hospitals in Ghana between January 2021 and September 2024. Head injury was defined as any traumatic injury to the scalp, skull, or intracranial tissues sustained during a road traffic crash among persons aged 0–18 years [8]. The severity of head injury was classified using the Glasgow Coma Scale (GCS) and the Head Abbreviated Injury Scale (Head AIS) [8]. The study included: (i) Patients aged 0–18 years who were diagnosed with head injury in the ED, and (ii) Patients with complete medical records containing demographics, pre-hospital time and mode of arrival, emergency vital signs, diagnosis, definitive trauma care, and discharge outcomes.

The study was conducted in three teaching hospitals: Tamale Teaching Hospital (TTH), Komfo Anokye Teaching Hospital (KATH), and Cape Coast Teaching Hospital (CCTH), located in the Northern, Ashanti, and Central Regions, respectively (Supplement Fig. 1). These centers have a bed capacity ranging from 400 to 1,200 and serve as referral hospitals and specialist training centers for clinicians and allied health professionals across Ghana’s three main ecological zones. These facilities are staffed by the country’s leading trauma and orthopedic surgeons, neurosurgeons, supported by multidisciplinary teams, along with associated health professionals, to ensure efficient 24-hour trauma care and emergency services for pediatric and adult accident victims [16, 17]. The study protocol was vetted and approved by the Institutional Review Boards of the three Teaching Hospitals.

### Sample size and sampling technique

The study required a sample size of 391 to estimate the proportion of pediatric RTA patients expected to experience unfavorable outcomes (death, vegetative state, and severe disability). The study assumed that 19% of patients presenting at the ED would have unfavorable outcomes [6] with a 95% confidence interval, 5% margin of error, and design effect of 1.5 to account for clustering within study centers. Using the Cochran’s formula, $$\:{(Z}^{2}\mathrm{*}P\left(1\mathrm{-}P\right)\mathrm{/}$$$$\:{e}^{2})\mathrm{*\:}\stackrel{\scriptscriptstyle\mathrm{def}}{=}.$$ A minimum sample size of 355 patients was calculated. To account for an anticipated 10% of records with incomplete or missing relevant data, the final sample size was adjusted to 391 patient records. Using monthly ED pediatric RTA admissions and probability proportional to size, 73 patients were allocated to CCTH, 147 to TTH, and 171 to KATH. The study employed a two-stage sampling method for the selection of study centers and pediatric patients. The sampling frame was all pediatric patients (18 years and below) who sustained road traffic accident-related injuries and reported to the three teaching hospitals. A detailed description of the sampling procedure was reported in a previous study by Baffour Appiah et al. [18].

### Data collection and measurement

We conducted a review of medical records and extracted data on patient biodata, crash and injury history, pre-hospital care, admitted services, medical and surgical intervention, inpatient complications, and clinical outcomes from the electronic medical records at the three centers. The type and form of electronic records were consistent across the three centers. The clinical records reviewed included neurologic assessment notes, patient discharge notes, neurological or occupational therapy notes, referral notes, and follow-up outpatient records. The review and extraction were guided using a pre-validated data extraction checklist. Our Trauma Registrars were trained on the study protocol, data collection procedure, and data validation process to ensure standardization. These activities were accompanied by biweekly in-process meetings to address any missing data and discrepancies in records.

Functional outcome following HI in this study was assessed using the modified GOS [19–21]. A standardized abstraction form with predefined operational definitions for GOS categories was used across all sites to improve consistency in retrospective outcome assessment. The section of GOS with adult-oriented items, such as occupational and independent social functioning, was replaced with pediatric discharge variables, including consciousness level, feeding ability, mobility, communication, developmental interaction, caregiver dependence, and discharge functional status (Supplement Table 1). Two Trauma Registrars at each site were trained on GOS assessment for pediatric patients, and data extraction was conducted under the supervision of trauma and orthopedic surgeons. Discrepancies in classifications were reviewed jointly during our biweekly meetings to achieve consensus based on available data, thereby minimizing inter-rater variability and outcome misclassification. The evaluation focused on key indicators, including HI-related symptoms, psychological status, recovery of consciousness, neuropsychological impairment, physical functioning, and survival. The GOS is a five-point ordinal scale that categorizes a patient’s functional outcome, including death, vegetative state, severe disability, moderate disability, and good recovery. The GOS was determined at the time of patient discharge.

The independent variables were classified into pediatric patient factors, pre-hospital factors, injury-related factors and inpatient factors. The pediatric patient factors included age, gender, and study center. The age was categorized as infants and toddlers (1–2 years), preschool (3-5years), school children (6–12 years), and adolescents (13–18 years). Pre-hospital factors comprised of mechanism of injury, mode of arrival at ED, transport time interval, and referral status. Mechanism of injury included pedestrian knockdown, motor vehicle crashes, motorcycle crashes, and tricycle crashes. The mode of arrival at ED was categorized as ambulance, private car, commercial cars, motorcycle and tricycles. Transport time was measured as the duration between time the accident occurred and time patient arrived at the tertiary facility in hours. Injury-related factors were presence of polytrauma, type of HI, injury severity score (ISS) and Glasgow Coma Scale (GCS). ISS categories adopted were mild (ISS < 9), moderate (9–15), severe (ISS 16–24) and critical (ISS > 24) [22]. Variables such as pediatric GCS score were categorized into three groups: normal/mild impaired conscious level (GCS 13–15); moderate (GCS 9–12) and severe (GCS 3–8) [23]. Inpatient factors were admission vital parameters and management modalities. Age-specific pediatric vital signs at presentation were extracted from patients’ medical records and reported as documented by attending clinicians. Vital parameters such as respiratory rate (RR), systolic blood pressure (SPB), diastolic blood pressure (DBP), heart rate (HR), oxygen saturation (SpO_2_), and temperature served as prognostic predictors. Principal management strategies included surgical and conservative interventions. The surgical interventions were classified as major and minor surgeries based on the World Health Organization (2009) Safe Surgery guidelines and the Ghana National Surgical, Obstetric, and Anaesthesia Plan (NSOAP) 2025–2029 framework [24, 25]. Major surgeries were defined as interventions involving the cranial cavity and/or requiring general anesthesia, such as craniotomy, decompressive craniectomy, evacuation of intracranial hematoma, and elevation of depressed skull fractures. Minor surgeries were defined as superficial interventions typically performed under local anesthesia, such as wound debridement, wound closure, and scalp laceration repair. Procedures were categorized in this manner because the study aimed to evaluate the association between surgical intervention and inpatient outcomes rather than the effects of specific surgical procedures. This study also included core specialty services such as neurosurgery, internal medicine, general surgery, orthopedics, and ophthalmology.

### Statistical analysis

The data were analyzed using STATA IC version 16 (StataCorp LLC 2019, College Station, TX, USA). Descriptive statistics such as frequency, proportion, mean (standard deviation), and median (interquartile range) summarized study variables. The Chi-square test was used to assess the difference in proportions of unfavorable outcomes among groups of categorical variables. A histogram was used to assess the normality of continuous variables. Mean difference was compared using the independent t-test (normally distributed), while median difference between groups was assessed using the Mann–Whitney U test (asymmetrical distribution).

The GOS was treated as discrete, ordinal data. However, one category (persistent vegetative state) had zero cases, and the overall distribution was skewed. Therefore, the ordinal scale was dichotomized into unfavorable outcomes (GOS ≤ 3) and favorable outcomes (GOS 4–5). This approach followed predefined, clinically relevant thresholds outlined in established guidelines [26, 27]. The outcome variable was coded as 1 as “unfavorable” and 0 as “favorable”. Clinically relevant covariates and independent variables that met the predefined p-value threshold < 0.25 from bivariate analysis were included in the multivariable logistic regression (Model 1), consistent with the procedure and methods from studies [8, 28, 29]. Variables with a Variance Inflation Factor (VIF) higher than 5 were excluded for multicollinearity [30]. The goodness-of-fit of the final logistic regression model was evaluated using the Hosmer-Lemeshow test. Subgroup analysis was performed for the three facilities that served as the study center to account for the facility effect. Significant odds ratios (OR) and 95% confidence intervals (CI) at *p* < 0.05 were presented with forest plots using R-studio (version 4.4.3).

The receiver operating characteristic (ROC) curve was used to evaluate the sensitivity, specificity, and overall discriminative performance of Model 1. This was further compared with a follow-up model (Model 2), which included only variables that remained statistically significant in Model 1. The estimated area under the ROC curve (AUC) was interpreted based on criteria for judging the existence of discriminative ability as used in a previous study [11]. Kaplan–Meier analysis was additionally performed to evaluate HI-related mortality according to Injury Severity Score and Glasgow Coma Scale categories.

## Results

During the study period, records of 1,602 pediatric RTA patients aged 0–18 years presented to the ED. Of these, 1,484 patients were included for a full review of head injury (HI) diagnoses, outcomes, and potential predictors. A total of 816 pediatric patients with HI, representing 54.95% of ED cases, were included in the final analysis. The flow chart of the study for pediatric RTA patients assessed for unfavorable outcomes of head injury is shown in Supplement Fig. 2.

**Fig. 1:**
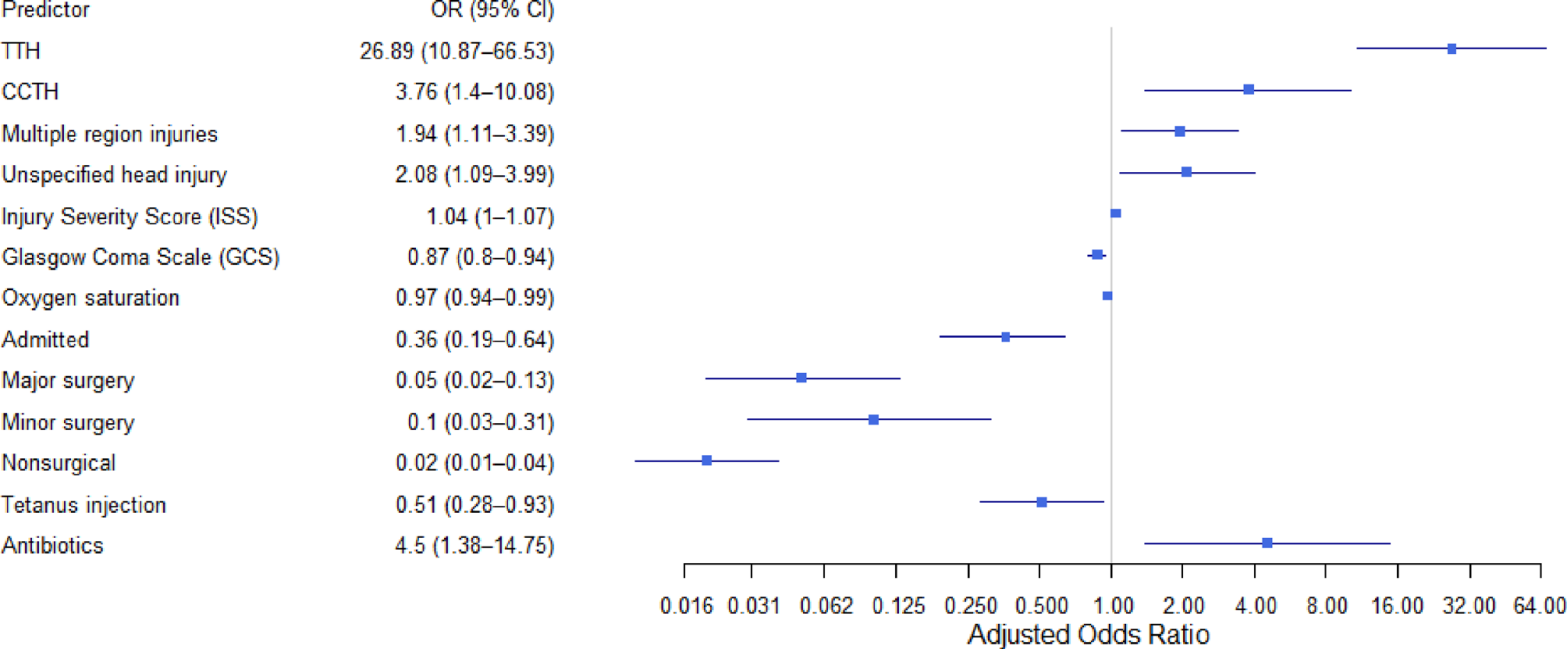
Predictors of unfavorable clinical outcomes of pediatric patients with head injury, Ghana, 2021–2024. TTH: Tamale Teaching Hospital, CCTH: Cape Coast Teaching Hospital, ISS: Injury severity scale, GCS: Glasgow Coma Scale

A total of 45 patients (5.51%) died in the hospital, 166 (20.34%) experienced severe disability, 281 (34.44%) had moderate disability, and 324 (39.71%) achieved good functional recovery at discharge. When grouped into binary outcomes, 211 patients (25.86%) had unfavorable outcomes, while 605 (74.14%) had favorable outcomes at discharge (Supplement Fig. 2). The median age was higher among those who died (12 years; IQR 4–15), while the median ages were equal among patients who experienced severe disability (10 years; IQR 5–16) and those who had a good recovery (10 years; IQR 6–16). The boxplot shows that children under 5 years were more likely to die than those in any other GOS category (Supplement Fig. 3).

There was no significant difference in mean age of patients with unfavorable outcomes (10.33 ± 5.61 years) compared with those with favorable outcomes (10.18 ± 5.60). The proportion of boys (25.9%) and girls (25.6%) with unfavorable outcomes was similar (*p* = 0.919). Among pediatric patients with HI, the most common mechanisms were pedestrian knockdowns (50.5%) and two-wheeled vehicle crashes (35.5%). However, three-wheeled vehicle crashes were associated with the highest proportion of unfavorable outcomes (41.7%) (*p* = 0.004). Patients transported via private vehicle (45.1%) and motorcycle (35.1%) were more likely to experience unfavorable outcomes. There was no significant difference in the mean transport interval between the two outcome groups (*p* = 0.120). The proportion of patients with unfavorable outcomes was significantly higher at TTH (46.0%) compared with the other study centers (*p* < 0.001) (Table 1).

**Table 1:** Prehospital characteristics of pediatric patients with head injury, Ghana, 2021–2024

Variables	Total patients (*n* = 816)^1^	Favorable outcome (*n* = 605)^1^	Unfavorable outcome(*n* = 211)^1^	*P*-value
**Age (years)**				
Mean ± SD	10.29 ± 5.60	10.33 ± 5.61	10.18 ± 5.60	0.735
**Gender**				0.919
Male	605 (74.1)	448 (74.1)	157 (25.9)	
Female	211 (25.9)	157 (74.4)	54 (25.6)	
**Mechanism of injury**				0.004
Pedestrian crash	412 (50.5)	316 (76.7)	96 (23.3)	
Two-wheeled crash	290 (35.5)	205 (70.7)	85 (29.3)	
Motor vehicle crash	66 (8.1)	56 (84.9)	10 (15.1)	
Three-wheeled crash	48 (5.9)	28 (58.3)	20 (41.7)	
**Patient referred status**				0.061
Direct entry	293 (35.9)	206 (70.3)	87 (29.7)	
Referral	523 (64.1)	399 (76.3)	124 (23.7)	
**Mode of arrival**				
Ambulance	370 (45.3)	298 (80.5)	72 (19.5)	< 0.001
Motorcycle	188 (23.0)	114 (60.6)	74 (39.4)	< 0.001
Private vehicle	91 (11.2)	50 (54.9)	41 (45.1)	< 0.001
Commercial vehicle	167 (20.5)	143 (85.6)	24 (14.4)	< 0.001
**Interval time (hours)** ^**a**^				
Mean ± SD	1.59 ± 1.45	1.64 ± 1.53	1.46 ± 1.18	0.120
**Study center**				< 0.001
KATH	305 (37.3)	293 (96.1)	12 (3.9)	
TTH	389 (47.7)	210 (54.0)	179 (46.0)	
CCTH	122 (15.0)	102 (83.6)	20 (16.4)	

SD: Standard deviation, ^a^Interval between the time of injury and time patient arrived at the teaching hospital. TTH: Tamale Teaching Hospital, KATH: Komfo Anokye Teaching Hospital, and CCTH: Cape Coast Teaching Hospital. ^1^Percentages in first column are column percentages, based on denominator of 816 to show distribution of variables. Percentages in second and third columns are row percentages, based on denominator of numbers in the categories of each variable, to show risk of unfavorable outcome

On descriptive (unadjusted) analysis, the most commonly diagnosed pediatric HIs were scalp injuries (37.6%), skull fractures (19.4%), intracranial hemorrhage (17.4%), and unspecified injuries (23.8%). In addition, 41.9% of patients sustained polytrauma. A significant proportion of patients with isolated HI (28.5%) experienced more unfavorable outcomes than those with polytrauma (22.2%) (*p* = 0.044). The median ISS did not differ significantly between the two groups (*p* = 0.166). However, patients with unfavorable outcomes had significantly lower median GCS scores than those with favorable outcomes (*p* < 0.001). Significant differences were observed in mean admission vital signs between the two outcome groups, except temperature (*p* = 0.877) (Table 2).

**Table 2:** Inpatient factors of unfavorable outcomes in pediatric patients with head injury, Ghana, 2021–2024

Variables	Total patients (*n* = 816)^1^	Favorable outcome (*n* = 605)^1^	Unfavorable outcome(*n* = 211)^1^	*P*-value
**Poly-trauma**				0.044
No	474 (58.1)	339 (71.5)	135 (28.5)	
Yes	342 (41.9)	266 (77.8)	76 (22.2)	
**Type head injuries**				
Scalp injuries	303 (37. 6)	252 (83.2)	51 (16.8)	< 0.001
Skull/basal injury	158 (19.4)	106 (67.1)	52 (32.9)	0.024
Intracranial hemorrhage	142 (17.4)	110 (77.5)	24 (21.8)	0.100
Cerebral contusion	40 (5.0)	29 (72.5)	11 (27.5)	0.827
Cerebral Edema	25 (3.1)	17 (68.0)	8 (32.0)	0.489
Diffuse Axonal Injury	34 (4.2)	24 (70.6)	10 (29.4)	0.645
Unspecified head injury^a^	192 (23.8)	99 (51.6)	93 (48.4)	< 0.001
**ISS score in the ED**				
Median (IQR)	9 (3–16)	9 (4–14)	9 (2–18)	0.166
**ISS score category in the ED**				0.026
Mild (≤ 8)	382 (46.8)	291 (76.2)	91 (23.8)	
Moderate (9–15)	212 (26.0)	166 (78.3)	46 (21.7)	
Severe (16–24)	152 (18.6)	102 (67.1)	50 (32.9)	
Critical (≥ 25)	70 (8.6)	46 (65.7)	24 (34.2)	
**GCS in the ED**				
Median (IQR)	15 (11–15)	15 (12–15)	14 (9–15)	< 0.001
***GCS*** **categor** ***y*** **in the ED**				< 0.001
Mild TBI (13–15)	577 (70.7)	452 (78.3)	125 (21.7)	
Moderate TBI (9–12)	146 (17.9)	104 (71.2)	42 (28.8)	
Severe TBI (3–8)	93 (11.4)	49 (52.7)	44 (47.3)	
**Vitals in the ED**, Mean ± SD				
Respiratory rate (cpm)	23.06 ± 5.70	22.72 ± 5.46	24.01 ± 6.25	0.005
Heart rate (bpm)	100.02 ± 26.29	98.14 ± 24.81	105.43 ± 29.57	0.001
Systolic BP (mmHg)	112.28 ± 19.40	110.52 ± 18.67	117.31 ± 20.60	< 0.001
Diastolic BP (mmHg)	67.04 ± 14.75	66.37 ± 14.80	70.04 ± 14.29	0.002
Temperature (^o^C)	36.72 ± 1.49	36.72 ± 0.63	36.74 ± 2.74	0.877
Oxygen saturation, Mean ± SD	96.58 ± 7.75	97.12 ± 6.09	95.07 ± 11.10	0.001

^a^Documented patient suffered moderate or severe head injury; IQR-interquartile range, *ISS-* Injury severity scale, *GCS*-Glasgow Coma Scale, TBI-Traumatic brain injury, SD-Standard deviation; ED- emergency department; BP, blood pressure, ^1^Percentages in first column are column percentages, based on denominator of 816 to show distribution of variables. Percentages in second and third columns are row percentages, based on denominator of numbers in the categories of each variable, to show risk of unfavorable outcome

The rate of unfavorable outcomes differed significantly by admission status (*p* = 0.002), with the highest proportion (34.6%) observed among patients who were managed only in the emergency department. Patients admitted under general surgery, orthopedic, and other services often presented with concomitant injuries in addition to head injury. These associated injuries required management by the respective specialty teams and were present among some patients who experienced unfavorable outcomes, as shown in Table 3. Most patients (73.0%) received conservative interventions. A significant proportion of patients who underwent surgical interventions (35.2% of major and 35.6% of minor) recorded unfavorable outcomes, possibly because these patients presented with more severe and complex injuries requiring surgical intervention. The proportion of unfavorable outcomes differed significantly among patients who received conservative care (*p* < 0.001) and among those who underwent major surgical procedures (*p* = 0.017). Patients discharged against medical advice (46.9%) had a significantly higher rate of unfavorable outcomes (*p* < 0.001), partly reflecting greater injury severity and/or a poorer prognosis at the time of discharge. Unfavorable outcomes also varied significantly across the different types of conservative management provided (*p* < 0.001). There was no significant difference in the median length of hospital stay between the two outcome groups (*p* = 0.489) (Table 3).

**Table 3:** Treatment outcomes of pediatric patients with head injury, Ghana, 2021–2024

Variables	Total patients (*n* = 816)^1^	Favorable outcome (*n* = 605)^1^	Unfavorable outcome(*n* = 211)^1^	*P*-value
**Inpatient status**				0.002
A&E only	188 (23.0)	123 (65.4)	65 (34.6)	
Admitted	628 (77.0)	482 (76.7)	146 (23.3)	
**Admission service**				
Internal Medicine	33 (4.1)	33 (100.0)	0 (0.00)	< 0.001#
General Surgery	13 (1.6)	11 (84.6)	2 (15.4)	0.368
Orthopedics intervention	268 (33.5)	185 (69.0)	83 (31.0)	0.032
Ophthalmology	24 (3.0)	18 (75.0)	6 (25.0)	0.885
Neurosurgery intervention	525 (65.7)	411 (78.3)	114 (21.7)	< 0.001
**Definitive Treatment regime** ^**a**^				
Major surgical intervention	108 (13.2)	70 (64.8)	38 (35.2)	0.017
Minor surgical intervention	45 (5.5)	29 (64.4)	16 (35.6)	0.126
Nonsurgical intervention	596 (73.0)	505 (84.7)	91 (15.3)	< 0.001
DAMA^b^	99 (12.1)	0 (0.0)	99 (100.0)	< 0.001#
**Medical management**				
Tetanus injection	282 (34.6)	162 (57.5)	120 (42.6)	< 0.001
Catheterization	58 (7.1)	24 (41.4)	34 (58.6)	< 0.001
Blood transfusion	57 (7.0)	29 (50.9)	28 (49.1)	< 0.001
IV fluids	722 (88.5)	519 (71.9)	203 (28.1)	< 0.001
Antibiotics	686 (84.1)	481(70.1)	205 (29.9)	< 0.001
Analgesia	730 (89.5)	524 (71.8)	206 (28.2)	< 0.001
**Length of stay (in days)**				
Median (IQR)	4 (2–8)	4 (2–8)	4 (1–15)	0.489
**Length of stay category**				< 0.001
<2 days	188 (23.0)	123 (65.4)	65 (34.6)	
2–8 days	425 (52.1)	352 (82.8)	73 (17.2)	
≥9days	203 (24.9)	130 (64.0)	73 (36.0)	

^a^ Do not add up to 100% some had received multiple interventions before been discharged, ^b^Discharge against medical advice did receive first aids at emergency but refused definitive treatment most surgical interventions, LoS- length of stay, #Fischer Exact test, ^1^Percentages in first column are column percentages, based on denominator of 816 to show distribution of variables. Percentages in second and third columns are row percentages, based on denominator of numbers in the categories of each variable, to show risk of unfavorable outcome

### Multivariable logistic regression model

Details of the unadjusted and adjusted regression model as shown in Supplement Table 1. In the multivariable logistic model, patients managed at TTH (AOR = 26.89; 95% CI: 10.87–66.53) and at CCTH (AOR = 3.76; 95% CI: 1.40–10.08) had significantly higher odds of unfavorable outcomes compared with those treated at KATH. In addition, patients with polytrauma (AOR = 1.94; 95% CI: 1.11–3.39) and those with unspecified head injuries (AOR = 2.08; 95% CI: 1.09–3.99) had higher adjusted odds of unfavorable outcomes. Each one-point increase in ISS (AOR 1.04, 95% CI 1.00–1.07) was associated with a 4% increase in the odds of an unfavorable outcome. Receiving antibiotics was associated with 4.50-fold higher odds of an unfavorable outcome at discharge. This likely reflects greater injury severity and the presence of complications among patients requiring antibiotic treatment, rather than a direct effect of antibiotics. In contrast, a unit increase in GCS, higher oxygen saturation at admission, ward admission, and trauma care interventions, such as major surgery, minor surgery, conservative care, and tetanus injection, were significantly associated with lower odds of unfavorable outcomes following HI. These suggest that interventions were more likely to optimize TBI-related outcomes, while the magnitude of the AOR may reflect either the effectiveness of each clinical intervention or the severity and complexity of injuries among patients who received them (Fig. 1).

### Subgroup analysis

**Fig. 2:**
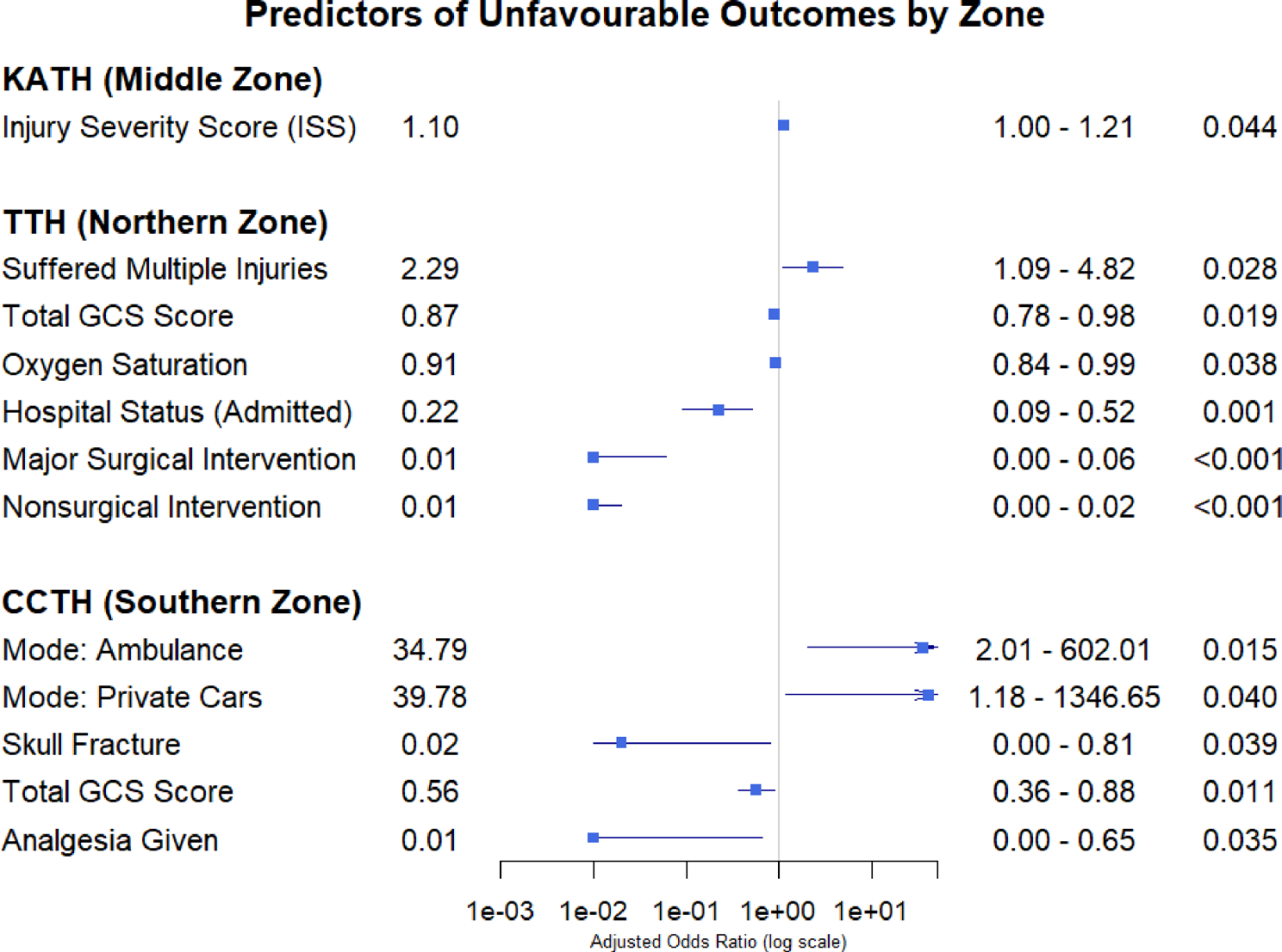
Stratified analysis of predictors of unfavorable outcome by study centers, Ghana, 2021–2024. KATH: Komfo Anokye Teaching Hospital, TTH: Tamale Teaching Hospital, CCTH: Cape Coast Teaching Hospital, OR: odds ratio, CI: confidence interval

We repeated the multivariable logistic regression for each facility. At KATH (Middle Zone), only ISS was statistically significant; a unit increase in ISS increased the odds of an unfavorable outcome by 10% (AOR 1.10; 95% CI: 1.00, 1.21) (Fig. 2).

At TTH (Northern Zone), six factors were significant: GCS score, polytrauma, oxygen saturation, admission status, and surgical interventions (minor/major). Patients with multiple injuries had 2.29 times higher odds of an unfavorable outcome (AOR 2.29; 95% CI: 1.09, 4.82). The remaining factors were protective, reducing the odds by 9–96%. Notably, a unit increase in GCS score reduced the odds by 13% (AOR 0.87; 95% CI: 0.78, 0.98) (Fig. 2).

At CCTH (Southern Zone), predictors included mode of transport, skull fractures, GCS score, and analgesic medication. Transport by ambulance or private car increased the odds of an unfavorable outcome by 34.79 and 39.78 times, respectively, compared to public transport. However, skull fractures, higher GCS scores, and receiving analgesics were associated with lower odds of unfavorable outcomes (Fig. 2).

### Sensitivity analysis

ROC analysis demonstrated excellent discriminative ability for both models. Model 1 achieved an AUC of 0.9107, while the reduced Model 2 achieved an AUC of 0.8955, indicating minimal loss in predictive performance after restricting the model to statistically significant predictors. However, Model 2 had relatively better sensitivity (77% vs. 76%), specificity (93% vs. 90%), and Youden index (0.70 vs. 0.66) (Supplement Fig. 4).

The Kaplan-Meier failure curve indicated a higher cumulative probability of HI-related mortality among pediatric patients with critical injuries (ISS ≥ 25) and severe injuries (ISS 16–24). Similarly, patients with severe HI (GCS 3–8) had a higher cumulative probability of mortality compared with the other GCS categories. The incidence of inpatient HI-related mortality differed significantly across ISS categories (*p* < 0.001) and GCS categories (*p* < 0.001) (Supplement Fig. 5).

## Discussion

Road traffic-related head injuries are a major contributor to global childhood injury mortality and disabilities, and are persistently higher in Sub-Saharan Africa [31]. This study examined the prehospital and clinical predictors of unfavorable outcomes of road traffic-related HI in children aged 0–18 years. The functional outcomes after HI were assessed using the Glasgow Outcome Scale, a standardized tool designed to estimate and monitor the psychological and physical functioning of patients with HI [6, 23].

In this study, the rate of unfavorable outcomes was 25.86% among pediatric patients with HI. This rate is comparable to 26.4% reported by Liu et al. [11] in China. Although Liu et al. [11] used the Glasgow Outcome Scale-Extended (GOSE), which differs from the original GOS in sensitivity and reliability, the comparable rates could be attributed to the uniform application of a modified GOS for pediatric patients in our study, incorporating similar predefined items to measure unfavorable outcomes, as well as the nature of trauma care provided at tertiary facilities. Although the death rate was low, a substantial proportion experienced severe or moderate disability, while nearly 40% achieved good recovery at discharge. A previous study using the GOS reported 11.7% mortality rate and 78% good recovery at six-month follow-up in both pediatric and adult populations [32], but relatively higher death rates were documented in previous studies in Malawi and Saudi Arabia [6, 33]. These disparities could be attributed to injury severity differences, level of prehospital and inpatient care, and underlying sociodemographic factors. Advancing trauma care in Ghana and similar LMICs requires a comprehensive strategy that includes training paramedics and bystanders in prehospital care, training clinicians based on Basic and Advanced Trauma Life Support principles [34, 35].

Findings from this study showed that extracranial injuries, such as skull fractures and scalp lacerations, were most the commonly reported injuries among patients. Unspecified injuries with diagnoses as “serious” and “severe” HIs were associated with higher odds of unfavorable outcomes. In this study, serious (or severe) HI referred to patients with a GCS score of ≤ 8, those requiring neurosurgical intervention or intensive care management [23]. The lack of documentation of exact HI among these patients could be due to their inability to afford a CT scan or to a faulty CT machine at the time of presentation, which hindered accurate detection of intracranial injuries. Although this study did not assess the association between access to CT and unfavorable outcomes, accurate CT diagnosis of brain injuries informs appropriate intervention and optimizes care [36]. On the contrary, the subgroup analysis revealed that skull fractures were associated with lower odds of unfavorable outcomes in southern Ghana. This finding may appear counterintuitive, as skull fractures are typically associated with severe head injury and resultant poor outcome [11, 37]. However, the observed deviation in our findings could be attributed to a lower (< 10%) proportion of patients reporting underlying intracranial injuries with skull fractures. This small subgroup size of patients with intracranial injuries, or injury heterogeneity, may have influenced the observed association.

The descriptive findings revealed that a higher proportion of patients with isolated HI experienced unfavorable outcomes compared to those with polytrauma. However, in the multivariable analysis, polytrauma was independently associated with increased odds of unfavorable outcomes after adjusting for potential confounders. In contrast to our findings, Liu et al. [11] reported a higher odds of unfavorable outcomes (OR = 1.33) among patients with isolated HI compared with those with polytrauma (OR = 0.97). However, this association was not statistically significant. Therefore, the evidence remains inconclusive and does not indicate that isolated HI predicts worse outcomes than polytrauma. The observed differences may be influenced by patient characteristics, injury severity, and clinical factors [11]. Therefore, the unadjusted findings should be interpreted with caution. Further research is essential to explore how clinical decisions and resource constraints, particularly in settings with limited access to advanced neuroimaging, affect functional outcomes in patients with isolated HI and polytrauma.

The relationship between GCS, ISS, and unfavorable outcomes such as deaths and disabilities has been studied [11, 33, 38]. A previous study suggests that lower GCS is associated with higher death rate and poor recovery rate [38]. These results are consistent with the final multivariable models for the Northern and Southern zones, where every unit increase in GCS was associated with a decrease in the odds of an unfavorable outcome. That suggests that a unit decrease in GCS is associated with a 15% increase in the odds of an unfavorable outcome. This study also found that an increase in ISS was associated with a 4% increase in the odds of an unfavorable outcome. This suggests that higher ISS is associated with an increased rate of deaths, complications, and poor functional recovery in children, especially those who sustain HI. Findings were consistent with a previous study, which reported that patients with ISS > 25 had higher rates of unfavorable outcomes [11]. Local interventions to improve helmet use and reduce the overall severity of traffic injuries in Ghana should be intensified, particularly in northern Ghana, where motorcycle crashes and HIs were most prevalent.

Pedestrian crashes and two-wheeled motor crashes were leading causes of pediatric HI, while three-wheeled crashes were associated with higher rates of unfavorable outcomes. These findings were comparable to reported higher rates of unfavorable outcomes and deaths among traffic accident victims [11, 32]. Children involved in vehicle knockdowns or two-wheeled vehicle accidents are at high risk of direct high-energy impact injuries, with limited head protection due to the lower rates of helmet use reported in study settings [8]. These increase the vulnerability of children to severe anatomical and neurological damage, and worsen prognosis [8, 39].

Our subgroup analysis highlights distinct regional variations, showing that patients managed at TTH and CCTH were 26.89 and 3.76 times more likely, respectively, to experience unfavorable outcomes after HI compared to those managed at KATH. Our data suggests that most injured patient referrals to TTH were motorcycle crash victims and reported a higher severity of HI, which were potential precursors of unfavorable outcomes [40]. Also, most patients transported by motorcycles notably resulted in unfavorable outcome due to poor handling of patients. However, the increasing risk of unfavorable outcomes among patients managed at CCTH could not be explained by study data. Potential reasons could be injury severity and in-hospital factors such as admission vital signs, and management modalities [7, 11].

The influence of admission vital signs as significant prognostic predictors of unfavorable outcomes has been reported in previous studies [11, 41, 42]. However, only oxygen saturation remained statistically significant in the final predictive model, particularly in the Northern zone. It was observed that a unit increase in the oxygen saturation was associated with a 3% reduction in the odds of an unfavorable clinical outcome after HI. Our finding corroborates with Egbohou et al., who found that patients with SpO2 < 90% had a twofold higher risk of death compared to those with SpO2 ≥ 90% [43]. Prompt emergency room assessment of SpO2 allows curative measures that are effective to minimize secondary injury and ultimately reduce morbidity and mortality [44].

Effective inpatient management of trauma patients is essential to enhance recovery and return to functionality [5, 45]. In this study, the majority of the patients presented to the facilities were admitted, 73.0% underwent conservative management, while 13.24% and 5.51% underwent major and minor surgical interventions, respectively. This pattern of care for trauma patients with HI was consistent with a previous study in India, which reported that more than 90% of patients underwent conservative management, while only 9.8% underwent surgical management [23]. Common conservative interventions received by the patients in the present study were consistent with recommended standard care for trauma patients, aimed at stabilizing the patient, preventing secondary brain injury or systemic failure, and optimizing recovery [5, 45]. The apparent discrepancy between the descriptive findings and the regression results on clinical interventions is likely due to differences between crude proportions and adjusted associations. After adjustment, trauma care interventions, including major surgery, minor surgery, and conservative care, were associated with lower odds of unfavorable outcomes. These findings suggest that although patients requiring surgical intervention were generally more severely injured, appropriate clinical interventions may still contribute to improved outcomes after head injury [5, 45].

The ROC curves are widely used to assess the discriminative ability of a measuring tool or predictive model, used in clinical settings [7, 11]. This study applied the ROC curve to evaluate the predictive performance of the multivariable predictive model for unfavorable outcomes in pediatric patients after HI, with a moderate sensitivity and high specificity of 69.52% and 94.81%, respectively. The findings are comparable to sensitivity and specificity reported in previous predictive models on HI outcomes that employed ROC curve [7, 11, 12]. This suggests that the model was better at ruling out unfavorable outcomes than detecting all patients at risk. However, AUC of 0.9107 for the model, represented an excellent discriminative performance of the model to distinguish between HI pediatric patients likely to have unfavorable versus favorable outcomes. However, identifying additional prehospital and inpatient variables in the multivariable predictive model could enhance the overall model accuracy.

This study provides valuable data to inform policy on pediatric head injury prevention and care in Ghana and similar LMICs. To the best of our knowledge, this is the first study to utilize multicenter clinical data to examine predictors of GOS among the pediatric population in Ghana, and one of only a few in Sub-Saharan Africa. Also, the study noted that patients’ inability to access a CT scan impeded accurate intracranial examination and increased the likelihood of unfavorable outcomes. Although the study was conducted across three major teaching hospitals, the sample may not fully represent all pediatric head injury cases in Ghana. Teaching hospitals tend to receive more severe or referred cases, which may introduce selection bias and limit the generalizability of the results to children treated at district or community-level facilities. Additionally, the study did not capture information on important patient-level factors such as underlying metabolic and endocrine disorders, nutritional deficiencies, or pre-existing neurological disorders, all of which may influence outcomes following HI. The predictive model demonstrated moderate sensitivity and an AUC below 1.0, indicating that while the model performed well overall, it did not perfectly discriminate between favorable and unfavorable outcomes. This suggests that other relevant prehospital, inpatient, or sociodemographic variables not available in the dataset may improve model performance. Also, the use of GOS in younger pediatric populations has recognized limitations because subtle neurodevelopmental deficits may not be fully captured, particularly in infants and toddlers. Furthermore, retrospective outcome assessment depended on the quality and completeness of clinical documentation. It may have introduced some degree of outcome misclassification despite the training of trauma registrars and the use of standardized abstraction procedures and review processes. Future research should employ larger, prospective, multilevel health facilities, incorporate serial follow-up assessments, and include additional clinical and contextual variables to enhance the accuracy and external validity of predictive models for pediatric head injury outcomes.

## Conclusions

There are significant regional disparities influencing the patterns of independent predictors of unfavorable outcomes in pediatric patients with HIs. Significant predictors of unfavorable outcomes included trauma center location, polytrauma, unspecified HI, ISS, GCS, oxygen saturation, major interventions, and tetanus injection.

Efforts to enhance diagnostic capacity and precision for HIs via standardized CT protocol and scale up specialized trauma care capacity, especially in the Northern Region, will reduce regional imbalances and risk of unfavorable outcomes. Future prospective, multi-level studies are needed to validate these predictors and guide the development of digital predictive models for integration into clinical practice and epidemiological research.

## Supplementary Information

Below is the link to the electronic supplementary material.

Supplementary Material 1

## Data Availability

The anonymized data are available on request via the Heidelberg University online data repository (the heibox) at [https://heibox.uni-heidelberg.de/f/33516a9fec974e4d860c/?dl=1] (https://heibox.uni-heidelberg.de/f/33516a9fec974e4d860c/?dl=1). All requests should be submitted to the corresponding author for review before access is granted.

## Abbreviations

CCTH: Cape Coast Teaching Hospital
GCS: Glasgow Coma Scale
HI: Head injury
ED: Emergency departments
GOS: Glasgow Outcome Scale
KATH: Komfo Anokey Teaching Hospital
ISS: Injury Severity Score
SPB: Systolic blood pressure
RR: Respiratory rate
DBP: Diastolic blood pressure
RTA: Road Traffic Accident
TTH: Tamale Teaching Hospital
ROC: Receiver operating characteristic
