# Supplementary material for: Pediatric road traffic accident – related head injury: a retrospective multicenter cohort study in Ghana
Source: BMC Emerg Med. 2026 Jul 18;26:197. doi: 10.1186/s12873-026-01687-5 (PMC13386704; doi:10.1186/s12873-026-01687-5)
Supplement: Supplementary file 1 — Supplementary Material 1 [file 12873_2026_1687_MOESM1_ESM.docx]

**Factors associated with unfavorable outcomes of pediatric road traffic–related head injury: a retrospective multicenter cohort study in Ghana**

Anthony Baffour Appiah^1*^, Till Bärnighausen^1^, Michael Lowery Wilson^2^, Peter Dambach^1^, Mahsa MohammadNamdar^3^, Alexis Dun Bo-ib Buunaaim^4,5^, Martin Morna^6^, Vincent Ativor^7^, Peter Donkor^8^, Charles Mock^9^,

**ONLINE SUPPLEMENTAL CONTENT**

**
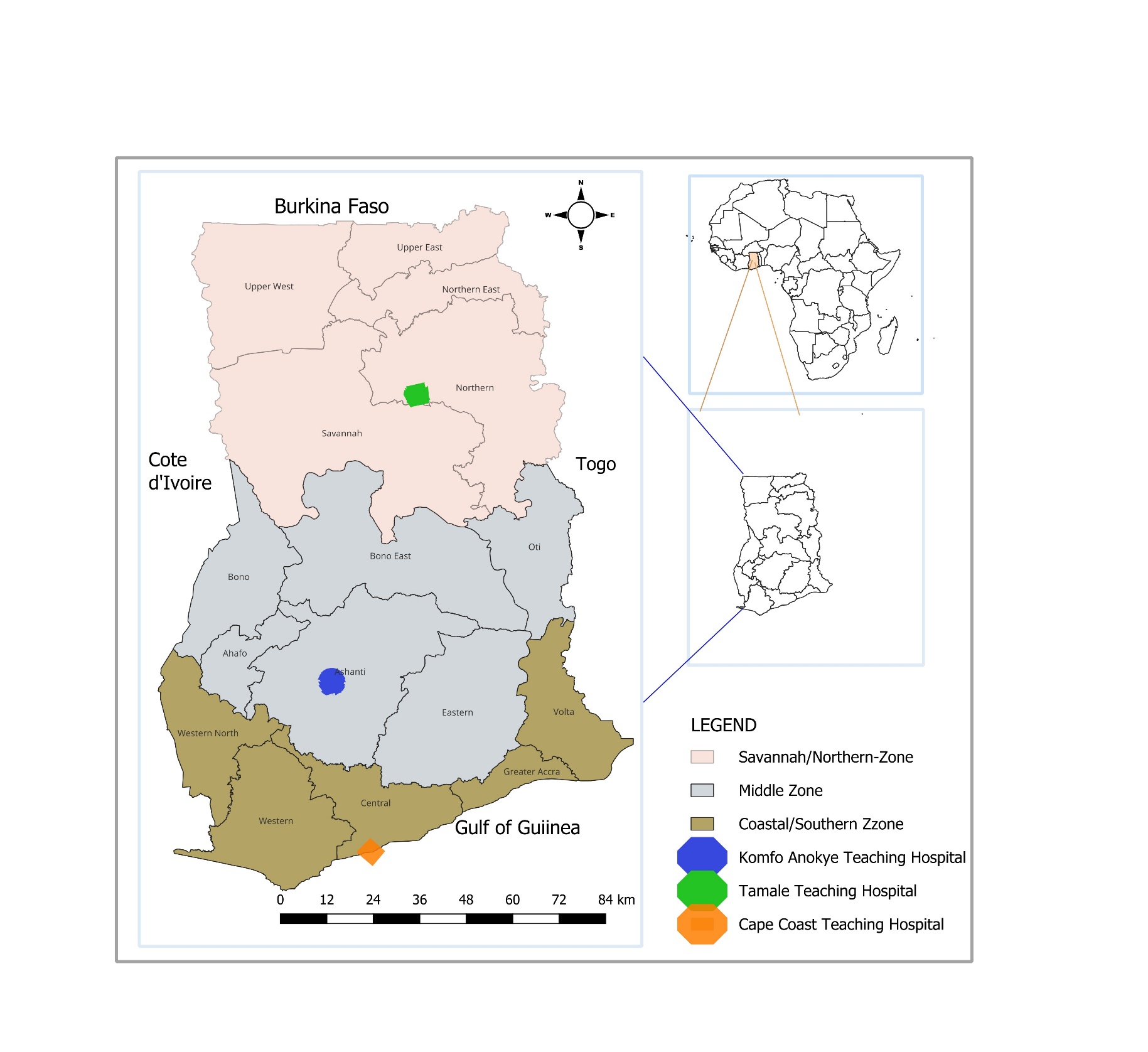
**

**Supplement Fig. 1.** Regional map of Ghana showing the three main zones and their teaching hospital serving as study sites

**
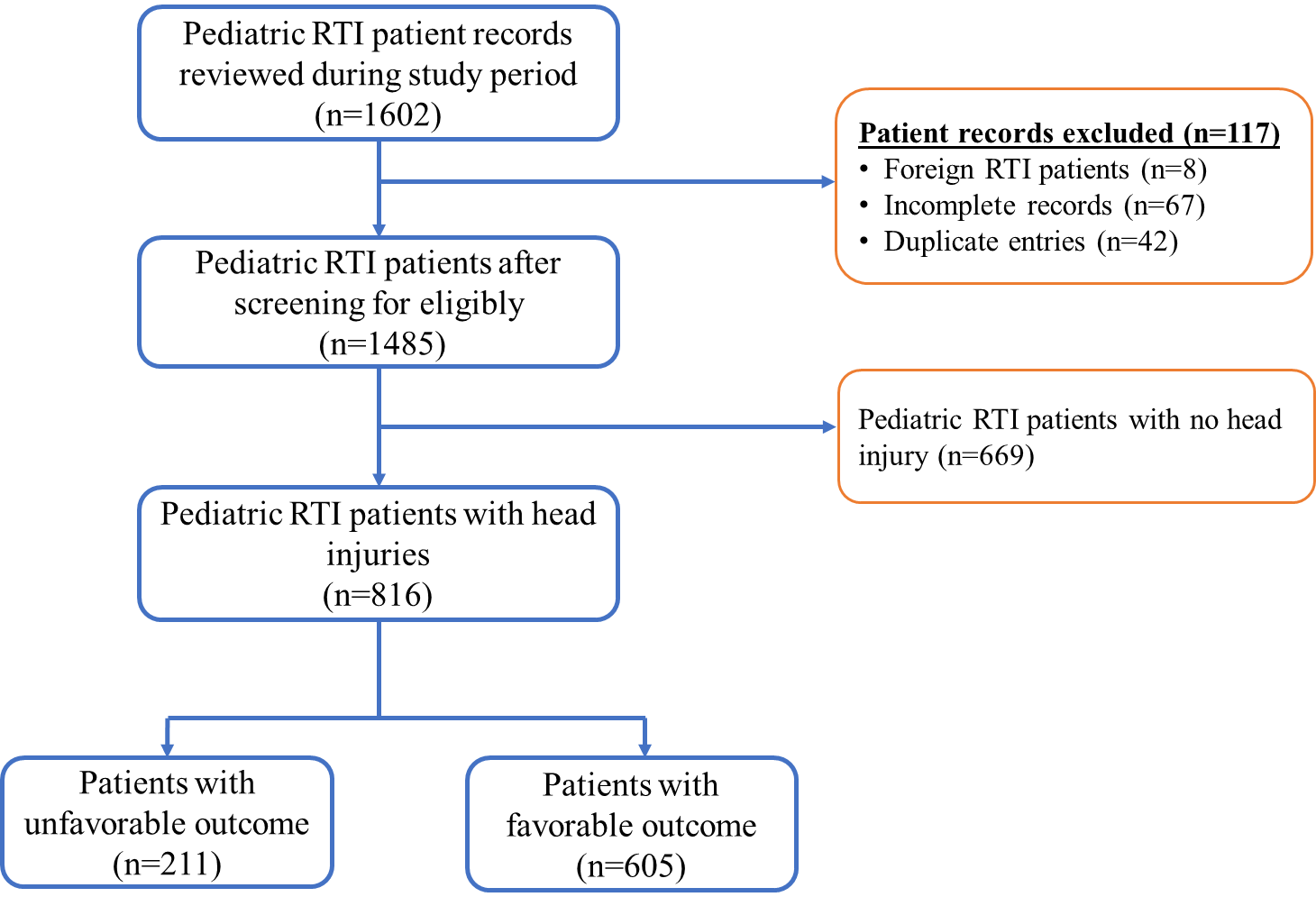
**

**Supplement Fig. 2.** Flow chart of the study for pediatric road traffic injury (RTI) patients assessed for unfavorable outcomes of head injury

**Supplement Table 1.** Predefined categories of retrospective pediatric GOS at discharge, pediatric road traffic study, Ghana

| **Definition** | **Tick as appropriate** |
| --- | --- |
| **GOS 5: Good Recovery** |  |
| Normal or near normal age-appropriate function |  |
| Independent feeding or mobility for age |  |
| Mild or no neurologic deficit |  |
|  |  |
| **GOS 4: Moderate Disability** |  |
| Conscious and independent |  |
| Persistent neurologic/functional deficits |  |
| Requires some assistance or rehabilitation follow-up |  |
|  |  |
| **GOS 3: Severe Disability** |  |
| Conscious but dependent |  |
| Severe mobility, feeding, or neurologic impairment |  |
|  |  |
| **GOS 2: Vegetative State** |  |
| Minimal awareness |  |
| Non-purposeful responses only |  |
|  |  |
| **GOS 1: Death** |  |
| Inpatient mortality |  |


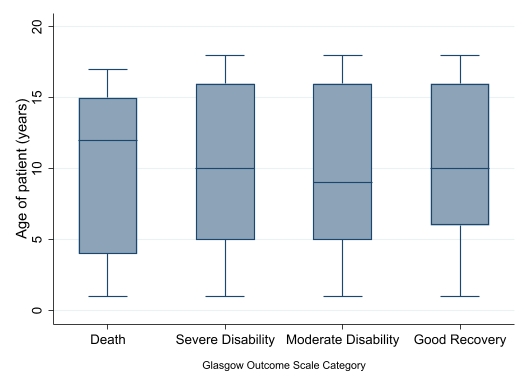


**Supplement Fig. 3.** Age pattern of pediatric Glasgow Outcome Scale in patients with traumatic head injury

**Supplement Table 2**. Predictors of unfavorable clinical outcomes of pediatric patients with head injury, Ghana, 2021-2024

| **Predictors** | **Crude OR (95%CI)** | **P-value** | **^a^Adjusted OR (95%CI)** | **P-value** |
| --- | --- | --- | --- | --- |
| **Mechanism of injury** |  |  |  |  |
| Pedestrian crash | Ref |  | Ref |  |
| Two-wheeled crash | 1.36 (0.97, 1.92) | 0.074 | 0.94 (0.54, 1.62) | 0.818 |
| Motor vehicle crash | 0.59 (0.29, 1.19) | 0.143 | 0.49 (0.18, 1.37) | 0.175 |
| Three-wheeled crash | 2.35 (1.27, 4.36) | 0.007** | 1.76 (0.70, 4.40) | 0.228 |
| **Patient referred status** |  |  |  |  |
| Direct entry | Ref |  | Ref |  |
| Referral | 0.74 (0.53, 1.01) | 0.062 | 1.01 (0.57, 1.79) | 0.974 |
| **Mode of arrival** |  |  |  |  |
| Ambulance (yes) | 0.53 (0.39, 0.74) | <0.001*** | 1.67 (0.79, 3.53) | 0.178 |
| Motorcycle (yes) | 2.33 (1.64, 3.29) | <0.001*** | 0.62 (0.28, 1.37) | 0.827 |
| Private vehicle (yes) | 2.68 (1.71, 4.19) | <0.001*** | 1.71 (0.76, 3.88) | 0.197 |
| **ED Arrival time interval** | 0.90 (0.78, 1.03) | 0.124 | 1.08 (0.91, 1.27) | 0.387 |
| **Study center** |  |  |  |  |
| KATH | Ref. |  | Ref |  |
| TTH | 20.81 (11.30, 38.33) | <0.001*** | 26.89 (10.87, 66.53) | <0.001*** |
| CCTH | 4.79 (2.26, 10.13) | <0.001*** | 3.76 (1.40, 10.08) | 0.009** |
| **Polytrauma** |  |  |  |  |
| No | Ref |  | Ref |  |
| Yes | 0.72 (0.52, 0.99) | 0.044* | 1.94 (1.11, 3.39) | 0.020* |
| **Type head injuries** |  |  |  |  |
| Scalp injuries | 0.44 (0.31, 0.63) | <0.001*** | 1.46 (0.84, 2.55) | 0.179 |
| Skull/basal injury | 1.54 (1.06, 2.24) | 0.025* | 1.00 (0.55, 1.83) | 0.988 |
| Intracranial hemorrhage | 0.53 (0.24, 1.14) | 0.106 | 0.92 (0.33, 2.58) | 0.988 |
| Unspecified head injury^a^ | 3.99 (2.83, 5.66) | <0.001*** | 2.08 (1.09, 3.99) | 0.027* |
| **Injury severity** |  |  |  |  |
| Injury severity scale score (ISS) | 1.03 (1.01, 1.04) | 0.001** | 1.04 (1.00, 1.07) | 0.028* |
| Glasgow Coma Scale (GCS) | 0.87 (0.83, 0.91) | <0.001*** | 0.87 (0.80, 0.94) | 0.001** |
| **Vital signs in the ED** |  |  |  |  |
| Respiratory rate (cpm) | 1.04 (1.01, 1.07) | 0.006** | 0.99 (0.95, 1.04) | 0.800 |
| Heart rate (bpm) | 1.01 (1.00, 1.02) | 0.001** | 1.00 (0.99, 1.01) | 0.405 |
| Systolic BP (mmHg) | 1.02 (1.01, 1.03) | <0.001*** | 1.01 (0.99, 1.03) | 0.192 |
| Diastolic BP (mmHg) | 1.02 (1.01, 1.03) | 0.002** | 0.98 (0.96, 1.01) | 0.164 |
| Oxygen saturation | 0.97 (0.95, 0.99) | 0.006** | 0.97 (0.94, 0.99) | 0.025* |
| **Inpatient status** |  |  |  |  |
| A&E only | Ref |  | Ref |  |
| Admitted | 0.57 (0.40, 0.82) | 0.002** | 0.36 (0.19, 0.64) | 0.001** |
| **Main intervention** |  |  |  |  |
| Major surgery (yes) | 1.68 (1.09, 2.58) | 0.018* | 0.05 (0.02, 0.13) | <0.001*** |
| Minor surgery (yes) | 1.63 (0.87, 3.06) | 0.130 | 0.10 (0.03, 0.31) | <0.001*** |
| Nonsurgical (yes) | 0.15 (0.11, 0.21) | <0.001*** | 0.02 (0.01, 0.04) | <0.001*** |
| **Medical management** |  |  |  |  |
| Tetanus injection (yes) | 3.61 (2.60, 4.99) | <0.001*** | 0.51 (0.28, 0.93) | 0.027* |
| Catheterization (yes) | 4.65 (2.69, 8.05) | <0.001*** | 1.51 (0.67, 3.42) | 0.319 |
| Blood transfusion (yes) | 3.04 (1.76, 5.24) | <0.001*** | 1.61 (0.70, 3.66) | 0.266 |
| IV fluids (yes) | 4.20 (2.00, 8.83) | <0.001*** | 2.69 (0.96, 7.57) | 0.059 |
| Antibiotics (yes) | 8.81 (3.82, 20.31) | <0.001*** | 4.50 (1.38, 14.75) | 0.013* |
| Analgesia (yes) | 6.37 (2.54, 15.94) | <0.001*** | 0.93 (0.24, 3.62) | 0.921 |

* Significance at p<0.05, **significance at p<0.01, ***significance at p<0.001, ^a^Model 1

**Supplement Fig. 4.** Receiver operating characteristic (ROC) curves for multivariable logistic regression models predicting unfavorable outcomes after head injury in the combined cohort. Model 1 included variables with P<0.025 in bivariate analysis, while Model 2 included only variables that remained statistically significant in the multivariable model. The discriminative performance was similar between the two models (AUC: 0.91 vs. 0.90). However, Model 2 had relatively better sensitivity (77% vs 76%), specificity (93% vs. 90%), and Youden index (0.70 vs. 0.66).

**
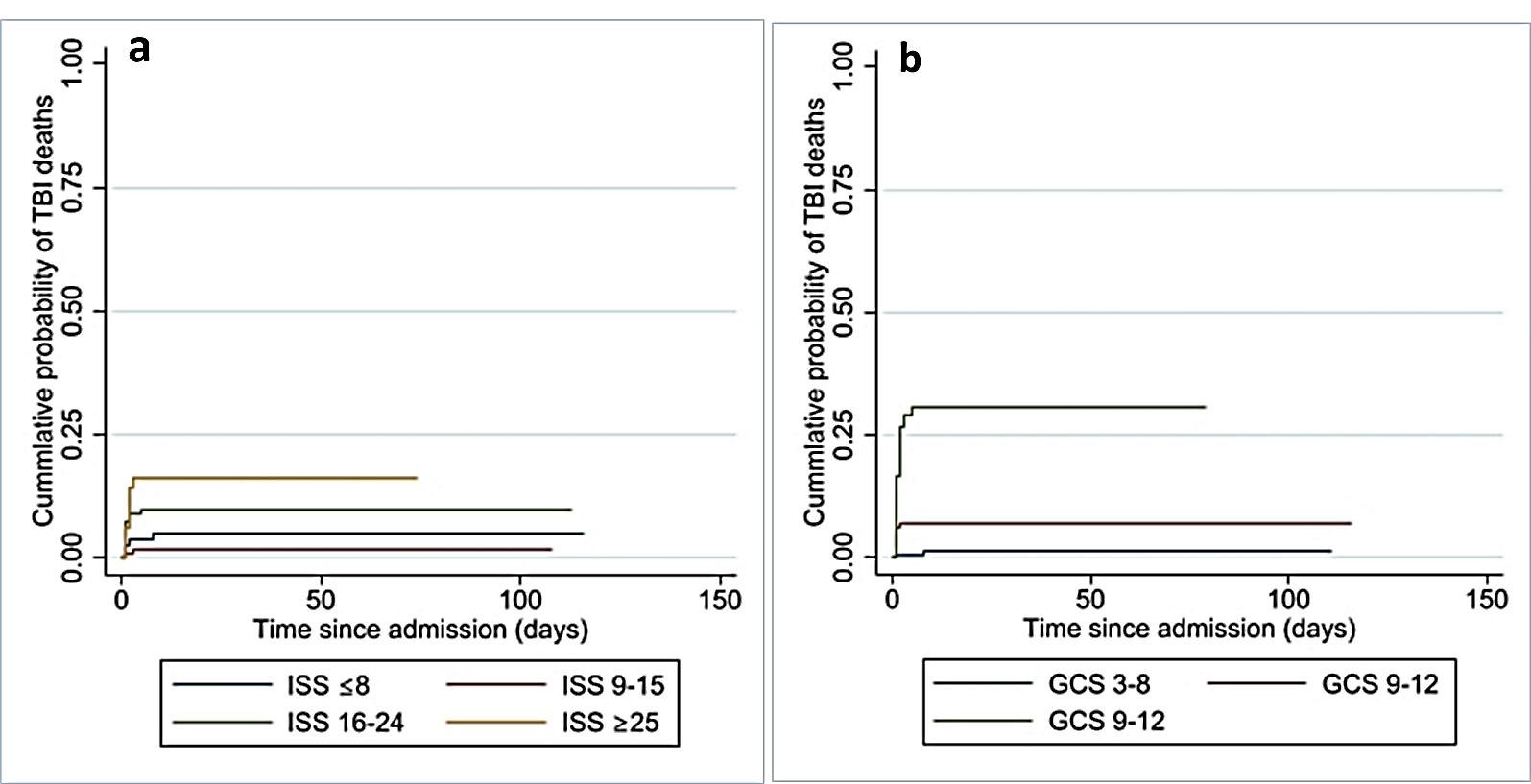
**

**Supplement Fig. 5.** Kaplan-Meier failure estimates of head injury-related mortality for Injury Severity Scale (3a) and Glasgow Coma Scale (4b) categories. In Fig. 4a, the cumulative mortality for Mild (≤8), Moderate (ISS 9-15), Severe (ISS 16-24), and Critical injury (ISS ≥ 25) is shown by the blue, red, green, and yellow lines, respectively. In Fig. 3b, the blue, red, and green lines show the pattern of cumulative mortality for GCS 13-15 (mild HI), GCS 9-12 (moderate HI), and GCS 3-8 (severe HI) groups, respectively**.**
